# Histopathological analysis of mucinous breast cancer subtypes and comparison with invasive carcinoma of no special type

**DOI:** 10.1038/s41598-021-85309-z

**Published:** 2021-03-11

**Authors:** Michał Piotr Budzik, Marta Magdalena Fudalej, Anna Maria Badowska-Kozakiewicz

**Affiliations:** 1 Department of Cancer Prevention, Medical University of Warsaw, 81 Zwirki i Wigury St, 02-091 Warsaw, Poland; 2 Doctoral School, Medical University of Warsaw, Warsaw, Poland

**Keywords:** Breast cancer, Cancer, Cancer, Breast cancer

## Abstract

Mucinous breast cancer (MBC) is a rare histological type of breast cancer characterized primarily by mucin's production and extracellular presence. MBC is usually associated with a better prognosis than other invasive breast neoplasms. Because of the low prevalence, MBC biology is not well understood. The aim of the present study was to introduce the last 2-year experience regarding MBC pathological diagnostics in our clinical center and comparison of the obtained data with invasive breast carcinoma of no special type (NST) comprising the most common invasive breast cancer. We identified 24 MBC cases representing 3.09% of all 766 invasive breast cancers, including 15 cases of pure type and 9 mixed MBCs. The median MBC patients' age at presentation was 65.5 years. Compared to NST, MBC presented a higher T stage with a statistically larger tumor median size, although lower regional lymph node involvement, tumor histological grade and TNM stage. MBC is a rare type of breast cancer, accounting for about 4% of all diagnosed breast cancers. Our findings are consistent with those published in recent years and show significant differences between MBC and NST cancer patients and also highlight differences between pure and mixed MBC, emphasizing the essence of their differentiation. MBC is associated with a better long-term prognosis than NST and is characterized by the less aggressive biological behavior expressed through favorable clinicopathologic features in terms of tumor grade, regional lymph node involvement and hormone receptor status.

## Introduction

Breast cancer is the most common invasive female cancer affecting around 14% of women and causing every year approximately 500,000 deaths worldwide. In fact, breast cancer is a heterogeneous group of neoplasms containing various cancers characterized by significantly different biology, clinical course and prognosis. The most common histological type of breast cancer is an invasive carcinoma of no special type (NST), previously known as invasive ductal carcinoma, not otherwise specified (IDC, NOS). NST is a group of cancers that does not present any specific differentiating features of other histological types of breast cancer. Among other breast cancers, we can distinguish numerous different types, often very rare, including mucinous breast cancer^1^.

Mucinous breast cancer (MBC), also named colloid carcinoma, is a relatively rare histological type of breast cancer, comprising about 4% of all breast cancer cases. However, pure type (PMBC) is even rarer and accounts for about 2%. MBC is characterized primarily by the production and extracellular presence of mucin^2^. According to the newest WHO classification of tumors of the breast (2019)^2^, MBC is classified as a special type of breast cancer and based on its cellularity, can be divided into two subtypes:

- The pure type (PMBC), which is composed entirely of tumor cells with extracellular and intracellular mucin in over 90% of the tumor mass and is more frequent;
- The mixed type (MMBC) which also includes infiltrating components such as ductal or lobular breast cancer-like and contains less than 90% of mucin^3^.

PMBC tumors may be classified as hypocellular (PMBC-A) tumors characterized by micropapillary, papillary, tubular, cribriform, or cordlike growth pattern and hypercellular (PMBC-B) tumors forming solid nests of cells floating in the mucin^4^. On the other hand, MMBC tumors may be subdivided into two groups based on the amount of mucinous component. According to Lei et al., it is possible to distinguish mMMBC tumors containing 50–90% of mucinous components and pMMBCs containing 30–50% of mucinous component^5^.

MBC is usually presented with a better prognosis than NST. Regional lymph node involvement and distant metastases are unusual findings^6,7^. Some MBCs, mainly mixed type, are associated with ductal and/or lobular neoplasia, while some present neuroendocrine differentiation. The etiology of MBC is multifactorial and involves common breast cancer risk factors; nevertheless, data suggests that some aspects related to reproductive events (e.g. late menarche, early menopause, childlessness) contribute less to MBC risk than to the risk of other histological breast cancer types^8^.

The aim of the present study was to introduce the 2-year experience of the Department of Pathology, Military Institute of Medicine in Warsaw regarding MBC pathological diagnostics and its comparison with NST cancers comprising the most common invasive breast neoplasm.

The article aims to revise the histopathological features of mentioned cancer subtypes (tumor size, presence of nodal involvement, the histological grade of malignancy, estrogen, progesterone and HER2 receptors expression) and to combine the above data with information on the cancer clinical course expressed as 5-year overall survival, 5-year disease-free survival and local recurrence rate. The present study's objective was to present MBC as a heterogeneous group of cancers, unlike numerous published reports, and answer the question about its prognosis compared to the most frequently diagnosed breast cancer subtype.

## Methods

The present study's material consisted of histological preparations derived from 776 women diagnosed with invasive breast cancer within the two years (2014–2015) in the Military Institute of Medicine, Warsaw. We evaluated demographic and clinical data and reviewed pathological findings of MBC and compared them to NST cancers. The material came from the patients who underwent surgery (modified radical mastectomies or breast-conserving surgery methods) and biopsies/excisional biopsies. Two independent pathologists evaluated tumor slides. Invasive carcinoma of no special type was found in 592 out of 776 patients (76.29%) and mucinous breast cancer was diagnosed in 24 cases (3.09%). Informed consent for participation in the study or use of their tissue was obtained from all participants. The median follow-up for the patients with breast cancer qualified for the study was 67.8 months (range 37–82).

Histological and immunohistochemical studies were done at the Department of Pathology, Military Institute of Medicine, Warsaw. Tumor samples were initially fixed in 10% phosphate-buffered formalin. After 24 h, fixation tissues were dehydrated in alcohol of gradually increasing concentration (50%, 60%, 70%, 80%, 90%, 96%) and subsequently by pure alcohol and xylene, and afterward embedded in paraffin. Paraffin blocks were cut into sections (4 µm). The sections were next stained with different methods for diagnostic purposes. Preparations stained with hematoxylin and eosin (H&E) were used to define the tumor histological type (WHO classification, 2019), the intensity of division (evaluating the degree of the mitotic index as the mean number of mitoses in cancer cells counted in 10 fields of vision at an objective magnification of 400 × (0.17 mm^2^ surface field) and histological grade of malignancy (G1–G3).

Constantly, all patients had a basic immunohistochemical profile assessed, i.e. expression of estrogen receptor (ER) and progesterone receptor (PR) and human epidermal growth factor receptor 2 (HER2). Immunohistochemical methods used paraffin sections attached to glass slides covered with 2% silane/acetone solution (Merck, Darmstadt, Germany) and dried for 24 h at 42 °C. Before commencing the immunohistochemical procedure, sections were dewaxed by inserting them in a series of alcohols of gradually decreasing concentrations, followed by washing in distilled water. Immunohistochemical assays were performed using the En-Vision + complex HRP DakoCytomatic (Dako, Santa Clara, United States) (En-Vision Dual Link System-HRP, DAB+, Code: K4065. Monoclonal antibodies against estrogen (Monoclonal Mouse Anti-Human Estrogen Receptor alpha, 1:50 dilution, Clone: 1D5, Code: IR654, Dako, Santa Clara, United States) and progesterone (Monoclonal Mouse Anti-Human Progesterone Receptor, 1:400 dilution, Clone: PgR636, Code: IR068, Dako, Santa Clara, United States) were applied to determine the expression of steroid receptors. The study was conducted as follows: histological sections were incubated in an incubator at 60 °C overnight and then dewaxed. The following step was to reveal the epitopes by heating slides in a buffer for 45 min. Afterward, preparations were left at 25 °C for 30 min. Preparations were rinsed in buffer, and then endogenous peroxidase was blocked in 3% hydrogen peroxide. In the following stage, preparations were incubated with appropriate antibodies. Afterward, sections were rinsed in a buffer for 15 min and then incubated with the visualization reagent for half an hour. Then the preparations were washed in Tris-Buffered Saline, pH 7.6, for 10 min, and then were incubated with 3,3ʹ-diaminobenzidine (DAB) (Substrate-Chromogen Solution) for 10 min to visualize the color reaction. Subsequently, color reactions were evaluated according to a scale regarding the extent and intensity of staining of nuclei in tumor cells. Nuclear staining in ≥ 10% of cancer cells was considered positive (+) for estrogen (ER) and/or progesterone (PR) receptor^9^.

Human epidermal growth factor receptor 2 (HER2) expression was determined using the HerceptTest Dako test (Code: K5204). It enables the HER2 detection using a polyclonal antibody against this protein (Rb A-Hu HER2—Rabbit Anti-human HER2 Protein). HER2 status was determined by assessing protein expression on the membrane of tumor cells using immunohistochemistry or evaluating the number of *HER2* gene copies using fluorescence in situ hybridization (FISH). The HER2 expression level was determined based on the maximum area of staining intensity as follows: strong circumferential membranous, staining > 30% of invasive carcinoma cells was graded 3 +, moderate, circumferential membranous staining in ≥ 10% of invasive tumor cells or strong circumferential membranous staining in > 30% of cells was designated as 2 + staining, weak and incomplete membranous staining in invasive tumor cells was marked as 1 + and no staining was scored 0. Score 0 and 1 + were considered negative for *HER2* amplification. Score 3 + was considered positive. Score 2 + was considered equivocal and FISH was ordered for confirmation. *HER2* was considered to be amplified if the average HER2 copy number was ≥ 6 signals/cells or ER2/CEP17 ratio ≥ 2^10^. Positive and negative control preparations were formerly performed.

All statistical analyses were performed with SPSS software v. 12.0 for Windows. The χ^2^ and Fisher's Exact Tests were used appropriately. Differences were considered statistically significant when p ≤ 0.05. The log-rank test was applied to compare the survival distributions and local recurrence rate.

### Ethics approval and consent to participate

The work described in this article has been carried out in accordance with the Code of Ethics of the World Medical Association (Declaration of Helsinki) on medical research involving human subjects; the ethical principles defined in the Farmington Consensus of 1997. The study was approved by the Bioethics Committee of the Medical University of Warsaw.

### Consent for publication

Informed consent for participation in the study or use of their tissue was obtained from all participants.

## Results

**Figure 1:**
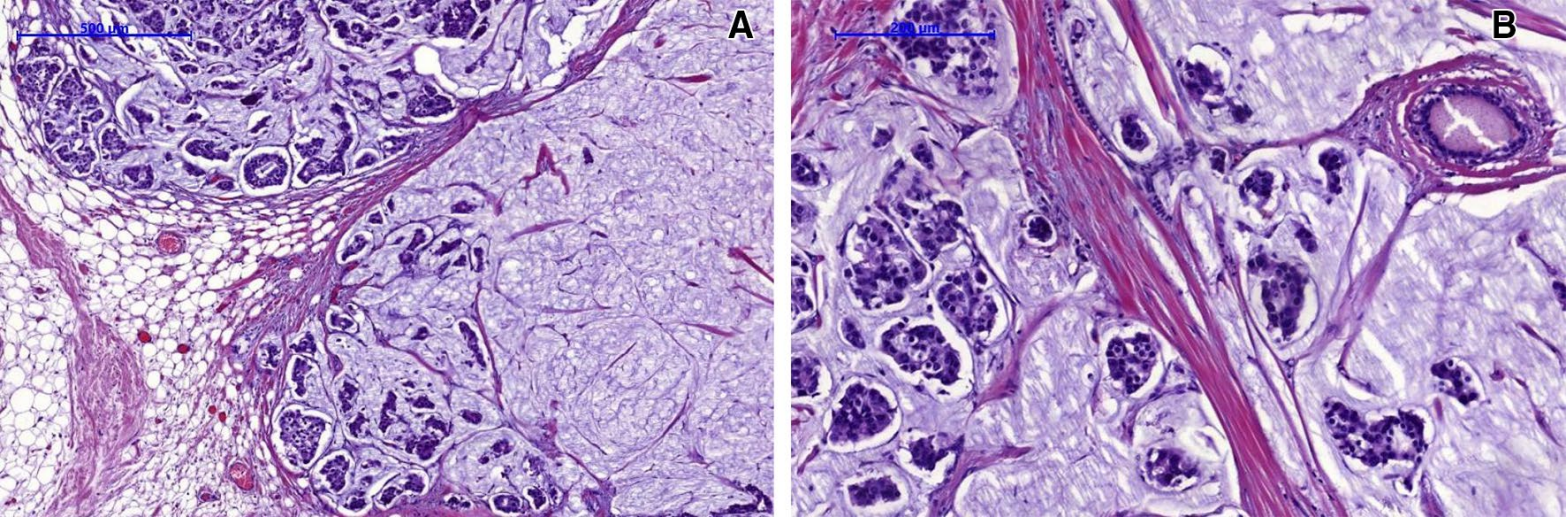
Histopathological image of pure mucinous breast cancer (PMBC) (grade 2, H&E)—characteristic cancer cells “nests” floating in abundant extracellular mucus (**A** low magnification; **B** high magnification).

**Figure 2:**
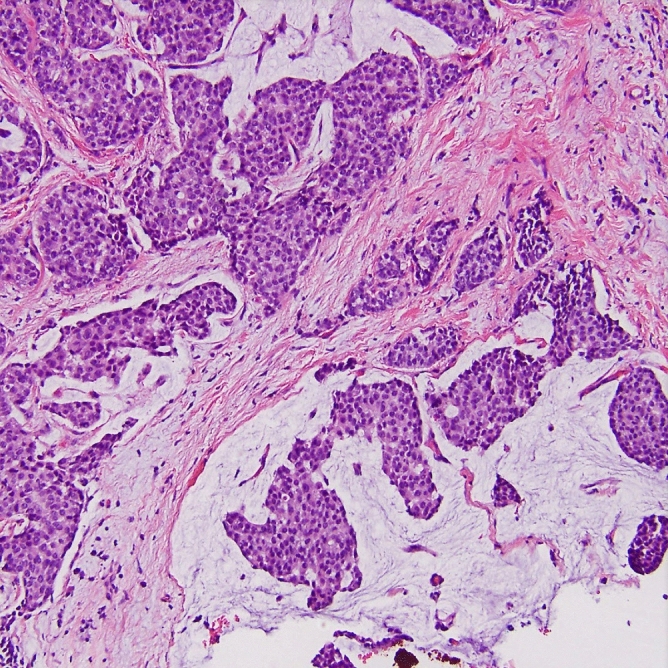
Histopathological image of mixed mucinous breast cancer (MMBC) with presence of invasive ductal carcinoma (IDC) component (grade 2, H&E, original magnification, 200 ×).

We identified 24 MBC cases representing 3.09% of all 766 invasive breast cancers diagnosed in our clinical center within two years (2014–2015) and compared obtained data with the characteristics of invasive carcinoma of no special type comprising the most numerous type of breast cancer (76.29% in the present study), hence, most often diagnosed (Table 1). In the group of mucinous breast cancer, we identified 15 pure mucinous breast cancers (PMBC) (Fig. 1) and 9 mixed mucinous breast cancers (MMBC) (Fig. 2; Table 2).

**Table 1:** Distribution of histological types in the group of 776 patients with invasive breast cancer.

Histological type of breast cancer	No	%
Invasive carcinoma of no special type (NST)	592	76.29
Invasive lobular carcinoma	98	12.63
Mixed ductal and lobular invasive carcinoma	32	4.12
**Mucinous (colloid) carcinoma (MBC)**	**24**	3.09
Tubular carcinoma	10	1.29
Metaplastic carcinoma	8	1.03
Carcinoma with medullary features	7	0.90
Invasive micropapillary carcinoma	5	0.81
	776	100.00

Statistically significant results *p* < 0.05.

**Table 2:** Comparison of pure and mixed mucinous breast cancer with NST cancer of the breast.

	MBC-total	PMBC	MMBC	NST
**Number of patients**	24	15	9	592
**Mean age at diagnosis**	66.0	64.1	69.7	60.0
**Patients aged over 80**	2	2	0	28
**OS 5-year (%)**	95.8%	100%	88.9%	75.3%
**DFS 5-year (%)**	91.6%	93.3%	88.9%	70.2%
**Local recurrence (%)**	8.3%	6.7%	11.1%	29.8%
**Median size**	2.19 cm	2.46 cm	2.01 cm	1.92 cm
**Tumor size (T) (no, %)**				
T1a	1 (4.2)	0	1 (11.1)	12 (2.0)
T1b	2 (8.3)	1 (6.7)	1 (11.1)	54 (9.1)
T1c	10 (41.7)	5 (33.3)	5 (55.6)	232 (39.2)
T2	9 (37.5)	8 (53.3)	1 (11.1)	248 (41.9)
T3	0	0	0	9 (1.5)
T4	2 (8.3)	1 (6.7)	1 (11.1)	37 (6.3)
**Nodal status (pN) (no, %)**				
pN0	21 (87.5)	14 (93.3)	7 (77.8)	354 (59.8)
pN1	2 (8.3)	1 (6.7)	1 (11.1)	142 (24.0)
pN2	1 (4.2)	0	1 (11.1)	63 (10.6)
pN3	0	0	0	33 (5.6)
**Tumor grade (no, %)**				
G1	0	0	0	46 (7.8)
G2	22 (91.7)	15 (100.0)	7 (77.8)	321 (54.2)
G3	2 (8.3)	0	2 (22.2)	189 (31.9)
Gx (necrosis/autolysis)	0	0	0	36 (6.1)
**Estrogen receptor status (no, %)**				
ER−	7 (29.2)	3 (20.0)	4 (44.4)	215 (36.3)
ER+	17 (70.8)	12(80.0)	5 (55.6)	377 (63.7)
**Progesterone receptor status (no, %)**				
PR−	9 (37.5)	4 (26.7)	5 (55.6)	241 (40.7)
PR+	15 (62.5)	11 (73.3)	4 (44.4)	351 (59.3)
**HER2 status (no, %)**				
HER2 0/1+	20 (83.3)	14 (93.3)	6 (66.7)	488 (82.4)
HER2 2+	0	0	0	32 (5.4)
HER2 3+	4 (16.7)	1 (6.7)	3 (33.3)	72 (12.2)

**Figure 3:**
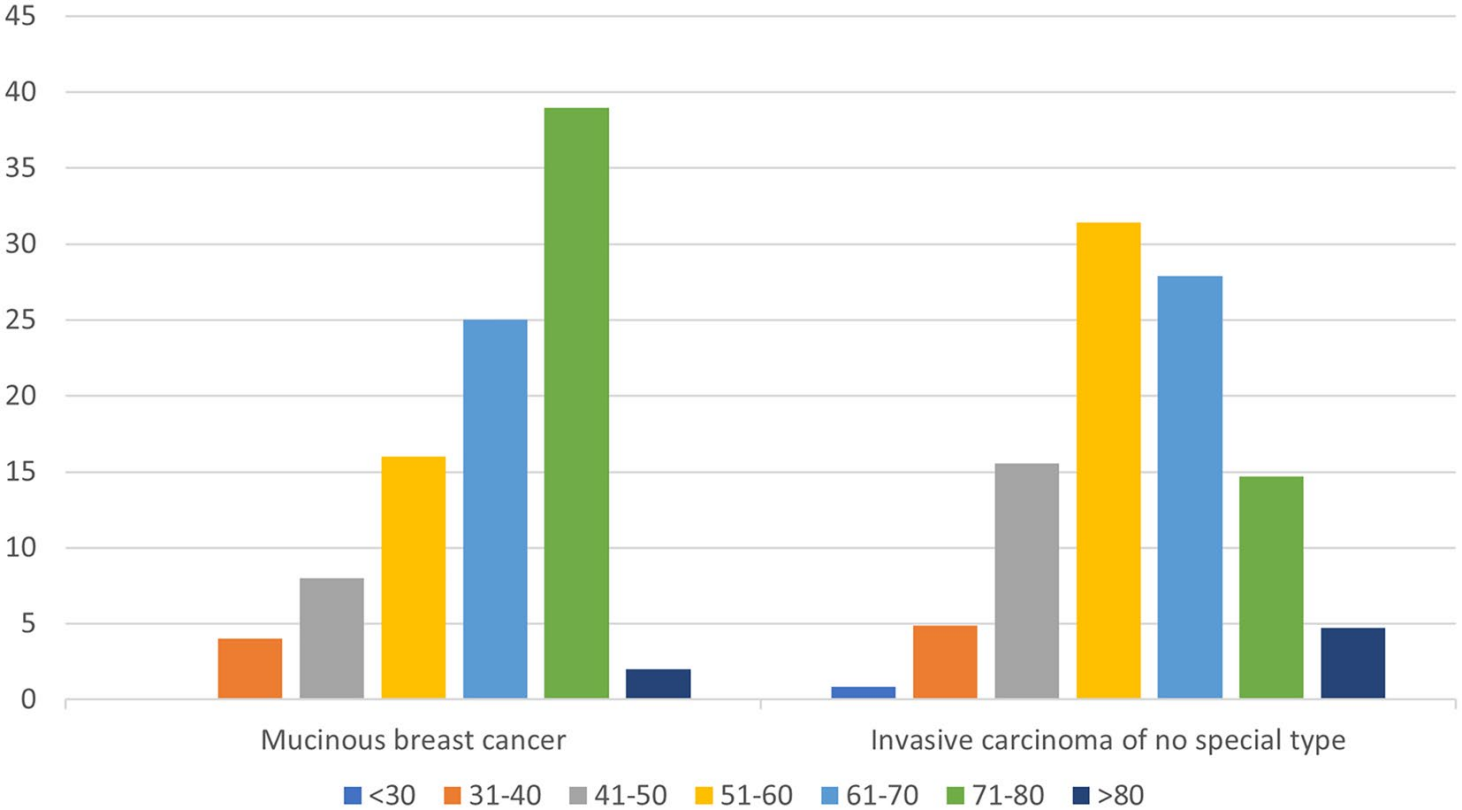
Age distribution of patients with mucinous and invasive carcinoma of no special type.

At the time of primary diagnosis, the vast majority (95.8%) of MBC patients were postmenopausal women. The median MBC patients' age at presentation was 65.5 (range 35–88) years versus 60.0 (range 27–91) for NST. It shows that MBC usually affects older women, especially in the postmenopausal period. The difference was statistically significant with p-value = 0.009. Comparing MBC subgroups, the mean age of PMBC patients was 64.1 years and 69.7 years of MMBC, respectively (Fig. 3, Table 2).

At the time of diagnosis, 83.3% of MBC patients presented a palpable mass in the breast, whereas the remaining 16.3% were diagnosed after the preventive mammography. The neoplasm's laterality was right-sided in 14 (58.3%) patients and left-sided in the remaining 10 (41.7%) women. All cases were unifocal.

Gross examination of MBCs showed a glistening, gelatinous lesion with pushing margins and fairly soft consistency. Typical of examined MBCs was mucoid material separated by usually fragile septa seen on the cut section. The majority of lesions were well circumscribed and bosselated. The tumors ranged in size from 0.7 cm to 4.0 cm (mean size 2.19 cm, PMBC mean size 2.46 cm; MMBC mean size 2.01 cm); tumor size was assessed as pT1 in 13 patents, pT2 in 9 patients and pT4 with 2 patients, with no pT3 cases. Only 3 (12.5%) patients presented regional lymph node involvement and only those who were diagnosed with MMBC (pN0—21 patients, pN1—2 patients, pN3—1 patient), while no distant metastases (M0) were identified.

In comparison to NST, MBC presented a higher T stage with a statistically larger tumor median size (2.19 cm for MBC and 1.92 cm for NST, p = 0.018). Moreover, patients with MBC were introduced with lower N stage (p = 0.007), lower tumor grade (p = 0.005) and lower TNM stage (p = 0.037). All these clinicopathological features are summarized in Table 3.

**Table 3:** Comparison of the clinicopathological features between mucinous breast cancer (MBC) and invasive carcinoma of no special type (NST).

	MBC		NST		p
	No. of patients	%	No. of patients	%	
**Age**					0.009
Median	65.5		60.0		
Average	66.0		60.0		
Range	35–88		27–91		
≤ 30	0	0.0	5	0.8	
31–40	1	4.2	29	4.9	
41–50	2	8.3	92	15.5	
51–60	4	16.7	186	31.4	
61–70	6	25.0	165	27.9	
71–80	9	37.5	87	14.7	
≥ 81	2	8.3	28	4.7	
**Median size**	2.19 cm		1.92 cm		0.018
**OS 5-year (%)**	95.8%		75.3%		0.013
**DFS 5-year (%)**	91.6%		70.2%		0.011
**Local recurrence (%)**	8.3%		29.8%		0.004
**Tumor size (T)**					0.748
T1a	1	4.2	12	2.0	
T1b	2	8.3	54	9.1	
T1c	10	41.7	232	39.2	
T2	9	37.5	248	41.9	
T3	0	0.0	9	1.5	
T4	2	8.3	37	6.3	
**Nodal status (pN)**					0.007
pN0	21	81.7	354	59.8	
pN1	2	8.3	142	24.0	
pN2	1	4.2	63	10.6	
pN3	0	0.0	33	5.6	
**Tumor grade**					0.005
G1	0	0.0	46	7.8	
G2	22	91.7	321	54.2	
G3	2	8.3	189	31.9	
Gx (necrosis/autolysis)	0	0.0	36	6.1	
**Estrogen receptor status**					0.474
ER−	7	29.2	215	36.3	
ER+	17	70.8	377	63.7	
**Progesterone receptor status**					0.925
PR−	9	37.5	241	40.7	
PR+	15	62.5	351	59.3	
**HER2 status**					1.000
HER2 0/1+	20	83.3	488	82.4	
HER2 2+	0	0.0	32	5.4	
HER2 3+	4	16.7	72	12.2	
**Stage (TNM)**					0.037
I	4	16.7	262	44.3	
II	9	37.5	224	37.8	
III	11	45.8	77	13.0	
IV	0	0.0	29	4.9	

Histological examination allowed us to identify 15 (62.5%) pure (PMBC, hypocellular/paucicellular variant) and 9 (37.5%) mixed (MMBC) mucinous breast cancers occurred usually with an invasive (ductal and/or lobular) or intraductal component noted mostly at the periphery of the tumor mass. One case with the presence of necrotic tissue in the central part of the tumor was found. Among MMBCs, there were 7 cases with an invasive ductal component, 1 with an invasive lobular component and 1 with the presence of intraductal non-invasive carcinoma. In situ component presented a micropapillary, papillary, tubular, or cribriform/comedocarcinoma pattern usually associated with prominent extracellular mucin production.

Typically, the microscopic features were individual epithelial cells or small clusters variable in shape with occasional tubular structure formation, floating in lakes of mucin subdivided by gentle fibrous septa containing fine capillaries. MBC cells usually present mild nuclear atypia, prominently visible nuclei, vesicular nucleoli and a moderate amount of cytoplasm. MMBC typically manifests neuroendocrine differentiation expressed as cytoplasmic argyrophilia and/or expression of neuroactivity markers: chromogranin, synaptophysin, neuronal-specific enolase. On the other hand, PMBC represents the typical non-endocrine variant^11^.

## Discussion

MBC is rarely seen in clinical practice, representing about 4% of all diagnosed invasive breast cancer. Despite the fact that many publications treat MBC as a homogenous group of cancers, in practice at least two subgroups (pure (PMBC) and mixed mucinous breast cancers (MMBC) differing in histology and clinical course might be distinguished. MBC prevails mainly in postmenopausal women and usually affects older patients compared to other invasive breast cancer types^12^ and is exceptionally rarely diagnosed in younger women under 35 years of age (1%)^7^. The mean age at diagnosis was 66 years compared to 60 years for NST patients. Considering MBC subtypes, PMBC affected younger women than MMBC (64.1 versus 69.7 years, respectively). Molecularly, MBC belongs most frequently to the luminal A subtype of breast cancer, characterized by the expression of genes typical for glandular cells that form the inner layer of normal ducts and lobules of the breast (inner luminal cells). Luminal A cancers show a strong expression of estrogen receptor (ER) genes and genes associated with its function regulation (*LIV-1*, *HNF3A*, *XBP1*, *GATA3*).

Compared to NST cancer, MBC presented with a higher T stage and hence by larger tumor size (2.19 cm vs. 1.92 cm, respectively), which was also reported by Park et al.^13^. Nevertheless, mucinous breast cancers are usually associated with smaller tumor size^5,14,15^. Although in our study MBC was presented with a larger tumor size than NST at the time of primary diagnosis, tumor size (T) appears not to be a significant independent factor associated with the severity of the disease because the mucin component comprises the majority of the tumor mass^16^. It stays in concordance with the American Joint Committee on Cancer (AJCC) staging system in which tumor size is not a significant factor in MBC^17^. It is presumed that in some cases, a large amount of mucin is answerable for hiding the disease until an extensive size is reached^18^. Some studies report tumor size as a prognostic factor, a less valuable one than nodal involvement^5,7,19^.

In the present study, MBC patients presented a higher hormonal receptor expression (ER, PR) and HER2 gene overexpression, which is untypical, but probably depends on a non-representative group of only four HER2-positive MBC cases reported. Previous studies also investigated that MBC was more often associated with steroid hormone receptors (ER, PR) expression, which is followed by the present results, in which the positive rates for ER and PR were 70.8% and 62.5% in MBC and 63.7% and 59.3% in NST, respectively^20,21^. The contemporary guidelines do not recommend chemotherapy or anti-HER2 therapy for hormone receptor-positive MBC, regardless of its HER2 status. In the study by Gwark et al. (2019), 11.8% of all 471 cases of PMBC were HER2-positive. Group of hormone-positive, lymph node-negative and HER2-positive cases with tumor size exceeding 3 cm presented worse disease-free survival (DFS) rates; thus, authors suggested a potential role of trastuzumab usage in this particular subgroup^19^.

Considering MBC subtypes, PMBC shows a higher T stage and a slightly lower N stage and tumor grade (G1-G3) compared to MMBC. PMBC seems to express steroid hormones receptors (ER, PR) more often than MMBC, which is reversed in HER2 expression. The results concerning nodal involvement stay in concordance with other studies' data, in which MMBC presented a greater capacity to metastasize^18,22^. On the other hand, in the investigation by Marrazzo et al. (2020), MMBC demonstrated a greater mass and higher T stage. What is worth mentioning, the obtained results were not statistically significant^18^. As far as hormonal status is concerned, the expression is suspected to be similar in both studied cases^22^; however, some studies suggest a higher incidence of luminal-A type among PMBC and higher incidence luminal-B type among MMBC^18^.

Diagnostic procedures pose some difficulties as MBC might resemble a benign lesion. The differential diagnosis could be challenging because MBC typically presents a round-shaped, well-circumscribed lesion, with misleading homogeneous isoechoic and normal posterior acoustic appearance at ultrasound examination^23^. At radiological diagnostics, MBC often shows non-mass mammographic findings, with missing calcifications or focal asymmetries. PMBC demonstrate less suspicious imaging features than cases of MMBC and could be mistaken for non-malignant breast lesions^24^.

Typically MBC is a slow-growing tumor and shows infrequently regional lymph node involvement; nonetheless, a metastatic disease worsens the survival rates and is regarded as the most significant prognostic factor^22^. Regional lymph node involvement and lymphovascular invasion were observed in the present study in the vast minority of MBC cases (12.5%), compared to NST (40.2%) (p = 0.007), which is comparable with other authors reporting the mean lymph node metastatic disease in 15% of MBCs^25^. It is also essential to distinguish MMBC from PMBC in terms of nodal involvement frequency (22.2% in MMBC versus 13.3% in PMBC), which was also observed in the previous studies, e.g. study by Marrazzo et al*.*, which reported nodal involvement in 31.58% of MMBC and 11.11% PMBC patients respectively^18^. Differentiation of PMBC from MMBC is also crucial since pure type has a better prognosis^26^.

It is also significant to search for additional microscopic features, such as micropapillary patterns, related to significantly worse overall prognosis^27^. A lobular component present in the tumor structure is usually associated with microscopic features of cell polarization loss, decreased cell to cell adhesion, and lack of neuroendocrine differentiation. Calcifications seen in conjunction with MBC often correspond to the invasive ductal component, and whenever visible, should prompt to search for this component. Also, neuroendocrine differentiation, defined by cytoplasmic argyrophilia and/or immunoreactivity to markers such as chromogranin, synaptophysin and neuronal-specific enolase, might be present within the tumor. Previous studies reported that neuroendocrine differentiation is associated with unfavorable 5-year and 10-year survival rates, overall survival and disease-free survival. Neuroendocrine differentiation is a poor prognostic indicator for mucinous breast cancer patients^28^.

Bearing in mind the mucinous biology of this type of breast cancer, it is worth mentioning that mucin 1 (MUC1) overexpression – a glycoprotein involved in the metastasis in various malignancies, was proved to worsen the prognosis of patients with breast cancer, including mucinous ones^29^. The possible mechanism might be correlated with the elevation of programmed death-ligand 1 (PD-L1) transcription by MUC1, and consequently, contributing to the immune escape of the aggressive forms of tumors^30^. The majority of PMBC overexpress the secreted mucin, mucin 2 (MUC2)^31^. Moreover, these cases are more resistant to chemotherapy, linked to the MUC2 overexpression^32^. Astashchanka et al. demonstrated that MUC2 plays an essential role in mediating the processes of apoptosis, proliferation, and metastasizing in breast cancer cells^32^.

MBC has a specific molecular identity different from NST^33^. Moreover, a lower genetic instability is a characteristic MBC feature, compared to NST and lobular breast cancers^6^. It was reported that almost all MBCs have a normal diploid stem line, unlike NST and lobular cancers. It has been proved that aneuploidy correlates with higher tumor grade (G) and stage (TNM)^34^.

MBC prognosis is more favorable compared to NST cancers. PMBC patients showed better overall survival (OS) and DFS rates than those for NST patients, but not significantly different from MMBC. MBC patients had a 5-year DFS rate of 91.6% (versus 70.2% of NST) and a 5-year OS of 95.8% (versus 75.3% of NST), which is similar to findings reported by Bea et al.^35^ and Cao et al.^13^. Some studies have reported that MMBC and NST patients are characterized by a significantly poorer prognosis than those with PMBC^7,36^.

Limitations of this study encompass a limited number of patients that might affect the achieved results. It brings us to another drawback that is the fact of a single-center study. Information on patients' long-term follow-up may also be enriching, but the collected data allow only for providing five-year indicators. The juxtaposition of results obtained in the different clinical centers would present a broader view of the discussed subject. Moreover, it is a retrospective study thus may present some potential selection bias. Nonetheless, we firmly believe that outcomes acquired in our center are a meaningful puzzle piece in the knowledge concerning MBC.

## Conclusions

MBC is a rare type of breast cancer, accounting for about 4% of all diagnosed breast cancers. Our findings are consistent with those published in recent years and show significant differences between MBC and NST cancer patients and also highlight differences between PMBC and MMBC, emphasizing the essence of their differentiation. Although it is crucial from the practical point of view and helps to predict the overall prognosis, there are still no tailored treatment strategies for MBC subtypes. Fortunately, all ones are characterized by a significantly better prognosis compared with NST patients. MBC is associated with a better long-term prognosis than NST and is characterized by the less aggressive biological behavior expressed through favorable clinicopathologic features in terms of tumor grade, regional lymph node involvement and hormone receptor status. Without any doubt, further research is needed to obtain a broader understanding of this breast cancer subtype's biology, resulting in even better survival rates.

## Data Availability

All data generated or analysed during this study are included in this published article.
